# Review of the Effects of Supplementary Cementitious Materials and Chemical Additives on the Physical, Mechanical and Durability Properties of Hydraulic Concrete

**DOI:** 10.3390/ma14237270

**Published:** 2021-11-28

**Authors:** Muralidharan Raghav, Taejoon Park, Hyun-Min Yang, Seung-Yeop Lee, Subbiah Karthick, Han-Seung Lee

**Affiliations:** 1 Department of Civil Engineering, PSG Institute of Technology and Applied Research, Neelambur, Coimbatore 641 062, India; raghav09psgitech@gmail.com; 2 Department of Robotics Engineering, Hanyang University, 55 Hanyangdaehak-ro, Sangrok-gu, Ansan-si 15588, Gyeonggi-do, Korea; taejoon@hanyang.ac.kr; 3 Innovative Durable Building and Infrastructure Research Center, Hanyang University, 55 Hanyangdaehak-ro, Sangrok-gu, Ansan-si 15588, Gyeonggi-do, Korea; yhm04@hanyang.ac.kr; 4 Department of Smart City Engineering, Hanyang University, 55 Hanyangdaehak-ro, Sangrok-gu, Ansan-si 15588, Gyeonggi-do, Korea; lsy871111@hanyang.ac.kr; 5 Department of Architectural Engineering, Hanyang University, 55 Hanyangdaehak-ro, Sangrok-gu, Ansan-si 15588, Gyeonggi-do, Korea

**Keywords:** supplementary cementitious materials, chemical additives, corrosion inhibition, special concretes, reinforcement corrosion

## Abstract

Supplementary cementitious materials (SCMs) and chemical additives (CA) are incorporated to modify the properties of concrete. In this paper, SCMs such as fly ash (FA), ground granulated blast furnace slag (GGBS), silica fume (SF), rice husk ash (RHA), sugarcane bagasse ash (SBA), and tire-derived fuel ash (TDFA) admixed concretes are reviewed. FA (25–30%), GGBS (50–55%), RHA (15–20%), and SBA (15%) are safely used to replace Portland cement. FA requires activation, while GGBS has undergone in situ activation, with other alkalis present in it. The reactive silica in RHA and SBA readily reacts with free Ca(OH)_2_ in cement matrix, which produces the secondary C-S-H gel and gives strength to the concrete. SF addition involves both physical contribution and chemical action in concrete. TDFA contains 25–30% SiO_2_ and 30–35% CaO, and is considered a suitable secondary pozzolanic material. In this review, special emphasis is given to the various chemical additives and their role in protecting rebar from corrosion. Specialized concrete for novel applications, namely self-curing, self-healing, superhydrophobic, electromagnetic (EM) wave shielding and self-temperature adjusting concretes, are also discussed.

## 1. Introduction

Cement has been the predominant material in the construction industry and is also one of the mostly used materials in the world, next to water [1]. The demand for Portland cement is increasing day-by-day, and thus the cement industry has increased production of cement. Meanwhile, CO_2_ emission footprint in the environment is mainly due to the production of cement, because the cement industry emits 850 kg of CO_2_ per ton of clinkers [2]. Therefore, SCMs have been used as cement replacement materials in consideration of the environmental factor. ASTM C125 [3] defines an admixture as a material other than water, aggregate, hydraulic cement, or fiber reinforcement used as an ingredient of concrete or mortar and added to the batch immediately before or during mixing. ACI committee 212 lists 20 important purposes for which admixtures are used [4], including to increase the plasticity of concrete without increasing the water content, to reduce bleeding and segregation, to retard or accelerate the time needed to set, to accelerate the early rate of strength development, to reduce the rate of heat evolution, and to increase the durability of concrete to specific exposure conditions

A great deal of research has been conducted with various SCMs and CA blended concrete. The performance of concrete can be augmented by adding various SCMs and CA in the concrete. Furthermore, most SCMs are industrial waste products, i.e., fly ash, (FA), rice husk ash, (RHA), ground-granulated blast-furnace slag (GGBFS), sugar cane bagasse ash (SBA), silica fume (SF), metakaolin (MK), etc. These SCMs contain SiO_2_ and Al_2_O_3_, which react with calcium hydroxide in the presence of moisture to give cementitious properties (ASTM C595) [5]. The addition of SCMs in the concrete not only minimizes the cement content, but also reduces costs and environmental pollution. The chemical additives (CA) are actually added to the concrete during mixing for multifunctional purposes which can include reducing the cost of concrete, achieving certain properties in concrete more effectively than by other means, and maintaining the quality of concrete during the stages of mixing, transporting, placing and curing in adverse weather conditions and to overcome emergencies during concreting operations. CA addition in concrete is able to protect rebar from corrosion in aggressive environments. Interestingly, with suitable CA addition, it is possible to make self-curing, self-healing, super hydrophobic, electromagnetic (EM), wave shielding and self-temperature adjusting concretes.

The main focus of this review is to demonstrate the effects of supplementary cementitious materials and chemical additives on the physical, mechanical and durability properties of hydraulic concrete. It aims to elaborate the effective utilization of SCMs (FA, GGBS, SF, RHA, SBA, and TDFA) and CA (corrosion inhibiting, self-curing, self-healing, super hydrophobic, electromagnetic (EM) wave shielding and self-temperature adjusting) on the distinct properties of concrete. This review is intended to the researchers to select those SCMs and CA most suitable for ordinary and hydraulic concrete thanks to their better physical, mechanical and durability characteristics. 

## 2. Supplementary Cementitious Materials (SCMs)

SCMs such as FA, GGBS, SF, RHA, SBA, and TDFA are typically added to concrete. Besides cost reduction and enhancement of the workability of fresh concrete, they can be successfully employed to improve the resistance of concrete to thermal cracking, alkali-aggregate reaction, resistance to chloride diffusion, and sulphate resistance [6,7,8,9]. ASTM has separate classifications covering natural pozzolans, fly ashes, GGBS, and others. ASTM C618 contains standard specifications for fly ash and raw or calcined natural pozzolan for use as an SCM admixture in Portland cement concrete [10]. The specification sets a limit on fineness, water requirements, pozzolanic activity, soundness, and chemical constituents. 

### 2.1. Fly Ash (FA)

**Figure 1:**
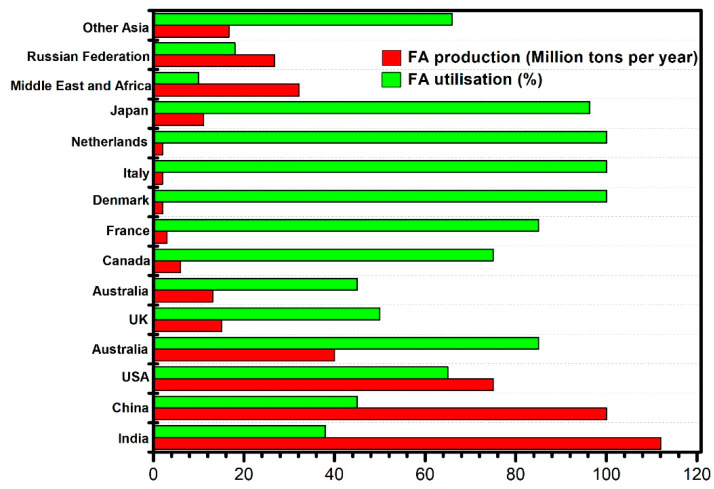
Worldwide production and utilization of fly ash and source data from [11].

Fly ash is a finely divided residue resulting from the combustion of ground or powdered coal. The worldwide production and utilization of FA is given in Figure 1 [11]. Fly ash is generally finer than cement and consists of glassy spherical particles. The use of fly ash in concrete began in the United States in the early 1930s. The major breakthrough research was conducted by Davis et al. [12] and Kohoku [13] by utilizing 120,000 metric tons of fly ash towards the construction of the Hungary horse dam in 1948. Less than 20% fly ash has been used in the cement industry during the last 30 years for pavement construction [14,15]. Significant strength gain was obtained beyond 28 days of curing [16]. ASTM C618 classified FA into two major classes, namely class ‘C’ and class ‘F’, based on chemical composition and type of coal burned. Class F fly ash is derived from the burning of anthracite or bituminous coal, while Class C fly ash is derived from the burning of lignite or sub-bituminous coal [17,18]. The chemical composition and physical properties of fly ash are given in Table 1. Class C fly ash usually has cementitious properties in addition to its pozzolanic properties due to free lime; on the other hand, Class F is rarely cementitious when mixed with water alone.

Specifications for fly ash are given in the ASTM C-618 [10] and AASHTO M 295 [19] standards. The United States transportation research council has prescribed norms and specifications [20]. Two properties, namely carbon content and fineness, will affect the air content and water demand of the concrete [21,22,23]. More fineness of FA demands higher water due to the increased surface area. The finer material requires more air-entraining agents, and the size of the particles is also important for a good mix design. Carbon content also affects water demand, since the carbon will absorb more water [24]. It is reported that fly ash mixed with Portland cement increases the volume of cementitious compounds when compared to non-fly ash concrete, as the paste volume is increased, leading to a reduction of aggregate particle interference and enhancing the concrete’s workability [25]. The shape of fly ash particles is spherical, which helps to improve their workability via the ball-bearing effect. This is because the spherically-shaped fly ash acts like tiny ball bearings during the mixing of concrete, which decreases the friction in a concrete mix, thus providing a lubricating effect which improves concrete workability [20,26,27,28].

Fly ash used in air-entrained and non-air entrained concrete mixes usually reduces the bleeding due to greater fineness volume and lower water content for a given workability [26,30]. Both types of fly ash were found to increase the setting time of concrete [20,30]; however, the setting time is influenced by the characteristics and amount of fly ash used in the concrete [26,31]. The strength of fly ash concrete depends on the type of cement used, quality of fly ash and curing temperature. For example, concrete containing class F may develop a lower strength at 3 or 7 days of curing when tested at room temperature [32]. In general, fly ash concrete gains strength with a longer curing period; however, the strength gain in cold weather conditions is more adversely affected when compared to non-fly ash concrete [33]. Therefore, it is necessary to take precautions when using fly ash in cold weather conditions [20]. Bouzoubaa et al. investigated the detrimental effect of fly ash concrete in deicer salt scaling conditions [34]. Thomas, conducted a review of field and laboratory studies on the resistance of fly ash concrete to salt scaling [35]. Lund et al. reported that concrete containing fly ash offers good resistance to freeze-thaw cycles [36].

Fly ash increases the cementitious compounds, minimizes water demand, and reduces bleeding channels, and thereby yields concrete with low permeable internal voids. Through the pozzolanic activity, fly ash chemically combines with water and calcium hydroxide, forming additional cementitious compounds which result in denser higher strength [37]. This result in reducing the amount of calcium hydroxide susceptible to attack by weak acids or other sulphates, and effectively reduces sulphate deterioration. Fly ash chemically binds with free lime in cementitious compounds, rendering it unavailable for sulphate reaction [38], which reduces concrete permeability and also reduces the amount of reactive aluminates, which are responsible for the sulphate reaction. Saca and Georgescu studied the behavior of C_3_A-rich cement containing fly ash in the presence of magnesium [39]. Kim et al., [40] and Mbessa and Pera [41] compared the performance of plain cement and fly ash-blended cement concretes for sulphate resistance by immersion in ammonium sulphate solution. They observed that fly ash-blended cement concretes have greater sulphate resistance than control concrete. Barbhuiya and Kumala [42] studied the sulphate resistance of Portland cement with fly ash and ultra-fine fly ash. The commercially available class F fly ash (FA) and ultra-fine fly ash (UFFA) were used as partial replacements in cement. The UFFA had 18% more amorphous content compared to FA. After 28 days of curing, the 100 mm cube samples were immersed in a 3% sulphuric acid concentration (H_2_SO_4_, pH≈3) and 1.5% nitric acid concentration (HNO_3_, pH≈3) for a period up to 90 days. In the sulphuric acid environment, the compressive strength loss was minimal for a concrete mix in which cement was replaced with 30% fly ash and 10% ultra-fine fly ash. The mass loss was less in this mix compared to the mix without fly ash. However, mass loss was also less in mixes containing higher amounts of fly ash. In the nitric acid environment, concrete mixes containing 20% fly ash and 10% ultra-fine fly ash and 30% fly ash and 10% ultra-fine fly ash had the minimum compressive strength loss. However, the mass loss in the mix containing 30% fly ash and 10% ultra-fine fly ash was less than the mix containing 20% fly ash and 10% ultra-fine fly ash [42].

#### Pozzolanic Reaction of Fly Ash 

**Figure 2:**
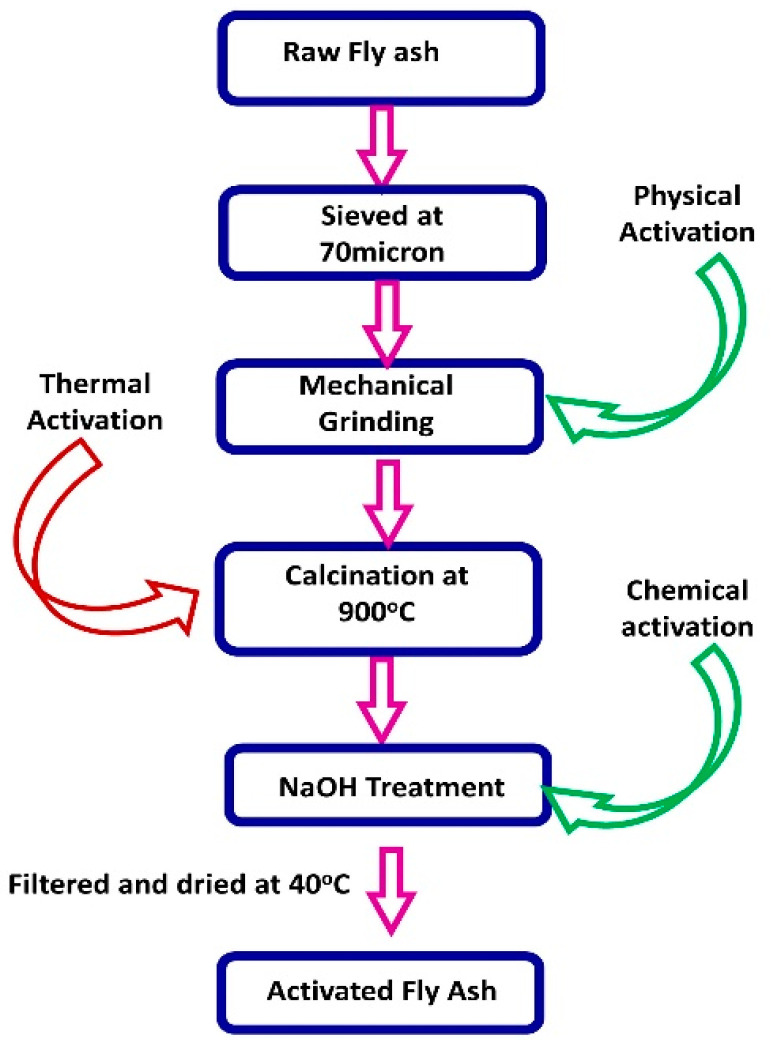
Schematic diagram for fly ash activation process.

**Figure 3:**
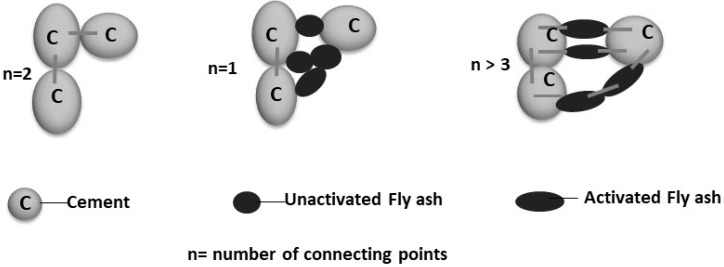
Binding mechanism of activated and inactivated fly ash.

FA needs activation due to two factors. First, the glassy surface layer of glass beads is dense, chemically stable and protects the inside constituents, which are porous, spongy and amorphous. Second, the silica–alumina glassy chain of high Si, Al, and low Ca is stable; the chain must be disintegrated if activity is to take place. The schematic diagram for the fly ash activation process is given in Figure 2. It is a fact that the presence of unburnt carbon and sulphur in the unprocessed fly ash may enhance the corrosion of reinforcement. Unburnt carbon content is an undesirable constituent of fly ash for use in reinforced concrete constructions. Besides its various harmful effects, it increases electrical conductivity. Because of the oxidizing atmosphere at power stations, the sulphur present in the fly ash is usually in the form of sulphates, which affect the spalling and disruption of concrete. The need for chemical activation of fly ash mainly involves the breaking of bonds and dissolution of the three-dimensional network structure of glass (Figure 3). It has also been reported that when Ca(OH)_2_ is present, the solubility of SiO_2_ in fly ash markedly increases. Thermal activation affects both fly ash reactivity and the kinetics of dissolution; significantly faster glass breakdown occurs at elevated temperatures by thermal activation. The binding mechanism of activated and inactivated fly ash is given in Figure 3. Based on the literature, fly ash up to 25 to 30% can be safely used to replace Portland cement with better properties. Activation of fly ash is essential in order to achieve better performance in both mechanical and corrosion-resistant properties. Activated fly ash cement was found to perform on par with ordinary Portland cement (OPC). The activated fly ash particles are smaller than cement particles, which can increase the degree of connection (Figure 3) and form inhomogeneous coagulation among cement particles, promoting cement setting. Fly ash is inert in the early period, and its connection with cement particles is weaker. The strong capability of CaO absorption by activated fly ash reduces the super-saturation degree in liquid resulting from early hydration of alite (3CaO·SiO). This can speed up alite hydration, meaning that hydrates of activated fly ash can behave as “crystal seeds” to promote the growth of C-S-H and Ca(OH)_2_, which is advantageous to coagulative structure formation.

### 2.2. Ground Granulated Blast Furnace Slag (GGBS)

**Figure 4:**
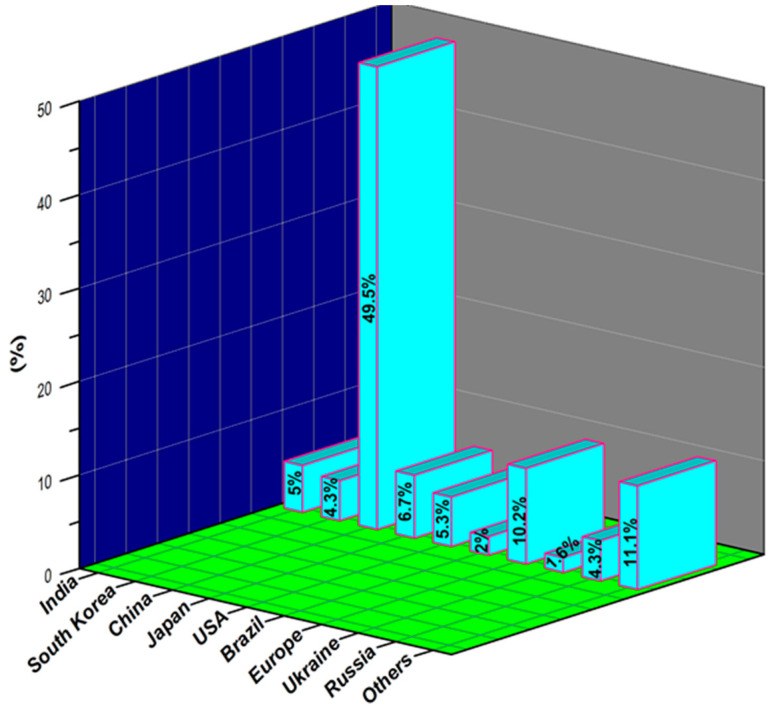
Worldwide production of GGBS and source; data from [43].

Ground granulated blast furnace slag is the granular material formed when molten iron blast furnace slag is rapidly chilled by immersion in water. The worldwide production of GGBS is given in Figure 4 [43]. The chemical composition of GGBS is given in Table 2. It is used in the form of granules with limited crystal formation, and its properties include a highly cementitious nature, fineness and ability to undergo a similar hydration process to Portland cement [20,44]. Two specifications, namely ASTM C 989-06 [45] and AASHTO M 302 [46], are in practice for the use of ground granulated blast furnace slag in concrete and mortar, with three grades, namely 80, 100 and 120.

GGBS is a cementitious material and can be substituted for cement on a 1:1 basis. The blast furnace cement is made by inter-grinding the granulated slag with Portland cement clinker. The grade of a GGBS is based on its activity index, which is the ratio of the cube compressive strength of a mortar made with 50% GGBS to a mortar made with cement [47,48]. The hydration activity index (HAI) of GGBS mortar can be calculated using the following Equation (1):

(1) HAI %=ftf0t×100

where

HAI = Hydration activity index of GGBS in %;

f_t_ = Compressive strength of test mortar at “t” age in MPa;

f_0t_ = Compressive strength of reference mortar at “t” age in MPa.

Grade 80 has a low activity index and generates less heat than Portland cement. Grade 100 has a moderate activity index, which is similar in cementitious behavior to Portland cement. Grade 120 has a higher activity index, which is more cementitious than Portland cement [49]. The use of slag cement usually improves workability and decreases water demand. It has been reported that the use of slag increases the paste volume caused by low relative density [50,51]. It has been reported that the compressive strength of slag concrete depends primarily upon the type, fineness, activity index and proportion of slag used in the concrete [49,52]. In general, slag-blended concrete develops lower strength at the initial curing period, i.e., 1–5 days, when compared to control concrete. However, a gradual increase in strength can be observed after 7 and 28 days [53,54]. The incorporation of GGBS in cement paste helps to transform the larger pores into smaller pores, resulting in a decrease in the permeability of the concrete [55]. It has been reported that this reduction of permeability is achieved due to the replacement level of slag increasing from 40 to 65% of the total cementitious material [56]. The reduction of permeability in concrete containing granulated slag may require less cover depth compared to conventional concrete to protect the reinforcing bars from corrosion. The effect on freeze-thaw durability of slag concrete has been studied by several researchers. It has been reported that the resistance of air-entrained concrete is comparable to that of conventional concrete [57,58,59]. Malhotra made a concrete containing 25–65% of GGBS with a different w/c ratio, and reported that the addition of GGBS improved the durability of concrete and satisfactorily increased its freeze-thaw resistance [60]. Bakharev et al. [61] and Khan and Sarker [62] studied concrete with partial replacement of Portland cement with alkali-activated GGBS. They reported that alkali-activated GGBS admixed concrete reduced the expansion of concrete when compared to control concrete, and that this is due to a low Ca/Si phase and high Al/Si phase that decreases the expansion of concrete produced with GGBS when compared to control concrete. 

#### Pozzolanic Action of GGBS

GGBS exhibits both glassy and crystalline phases. The SiO_2_ and CaO content in GGBS is 10–27% and 35–45% respectively. Hence, the active ingredients in GGBS readily react with cement and undergo hydration reactions, resulting in the formation of so-called C-S-H gel, which imparts strength to the concrete and leads to a pore blocking effect. Due to the inherently cementitious and pozzolanic nature of GGBS, it does not require any external activation methodology to increase the number of connecting points, as fly ash does. However, GGBS has undergone in situ activation with other alkalis present in it. In pore solution chemistry, GGBS endures both consumption and generation of calcium hydroxide during hydration reactions. However, GGBS favors the formation reaction rate, and hence undergoes self-activation, which leads to the usage of GGBS in high-performance concrete.

### 2.3. Silica Fume (SF)

Silica fume has been widely used as a supplementary cementitious material in high-performance concrete. Silica fume is also known as “micro-silica”. It is a by-product obtained from the reduction of high purity quartz with coal in electric furnaces during silicon and ferrosilicon alloy production. Silica fume is also collected as a by-product in the production of other silicon alloys such as ferrochromium, ferromanganese, ferromagnesium and calcium silicon [63]. Silica fume is generally available in two forms, dry and wet [64]. Dry silica is stored in silos and hoppers, while wet products are stored in tanks. The chemical composition of silica fume is given in Table 3 [65,66,67,68,69,70]. 

Silica fume consists of very fine vitreous particles with a surface area on the order of 20,000 m^2^/kg, which is approximately 100 times smaller than the average cement particle. Due to its extreme fineness and high silica content, it is effectively used as a pozzolanic material [71,72]. Mohamed and Mohamed reported that the addition of silica fume in different ratios led to improved mechanical properties in concrete. Further, electrochemical studies on steel embedded in silica fume-added concrete showed the lowest current density, indicating the better corrosion resistance of silica fume concrete [73]. AASHTO and ASTM C1240 [74] standards cover microsilica for use as an SCM in Portland pozzolana cement (PPC) and mortar to reduce the amount small voids and pores. Silica fume satisfies both the physical and chemical requirements of concrete and yields a good finish. The addition of silica fume to the concrete itself increases water demand; it requires one additional pound of water for every pound of silica fume added. This can be overcome by using high range water reducing agent (HRWR). It has been reported that with the addition of more than 10% silica fume, the concrete becomes sticky, which enhances workability and increases initial slump [75,76]. Silica fume reduces bleeding because of its rheological properties. The use of silica fume in concrete produces very high strength and low permeability concrete which is also chemically resistant [77,78]. It has been reported that the modulus of rupture is usually the same or higher than that of conventional concrete at the same level of compressive strength [79,80]. The air void stability of concrete incorporating silica fume was studied by Abbas et al., [81] and Karakurt and Bayazit [82], showing that the use of silica fume had no significant influence on the production and stability of the air void system or freeze-thaw durability. It has been shown by several researchers that the addition of silica fume to concrete reduces permeability [71,83]. Rapid chloride permeability tests (AASHTO 277) conducted on silica fume concrete revealed a significant reduction in chloride permeability (8% silica fumes) due to the increased density of the matrix in the presence of silica fume.

#### Influence of Silica Fume in Cement Matrix

**Figure 5:**
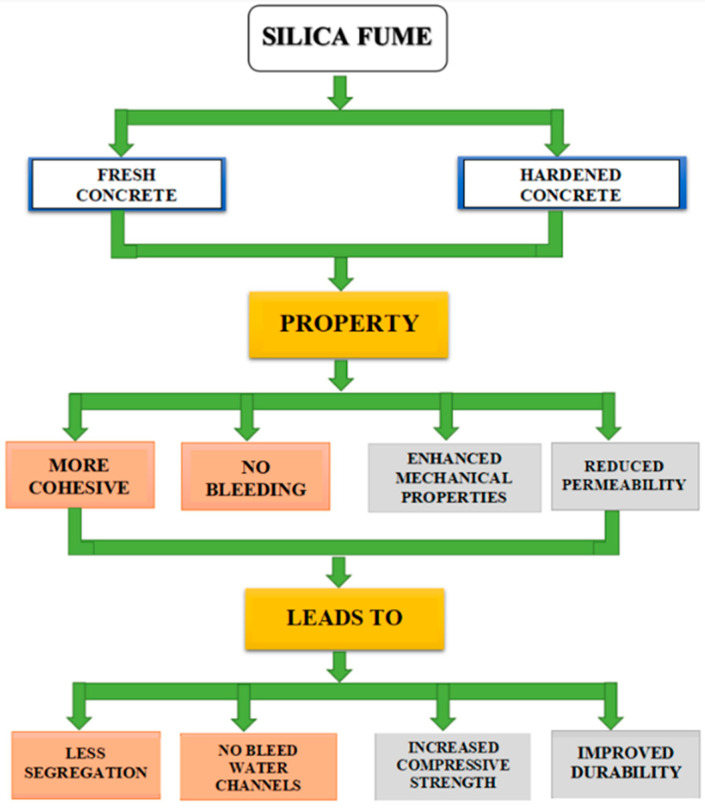
Schematic of mechanistic action of SF in fresh and hardened concrete.

The reaction of silica fume in concrete involves both a physical contribution and chemical action. As a physical contribution, adding silica fume fills the spaces in the interfacial region of the cement grains. This phenomenon is called particle packing. As a chemical contribution, silica fume contains more than 90% very highly amorphous SiO_2_. Thus, it is a highly reactive pozzolanic material and readily reacts with calcium hydroxide to form C-S-H gel and provide hardened concrete. The mechanism of action of silica fume in fresh and hardened concrete is given in Figure 5.

### 2.4. Rice Husk Ash Admixed Concrete (RHA)

**Figure 6:**
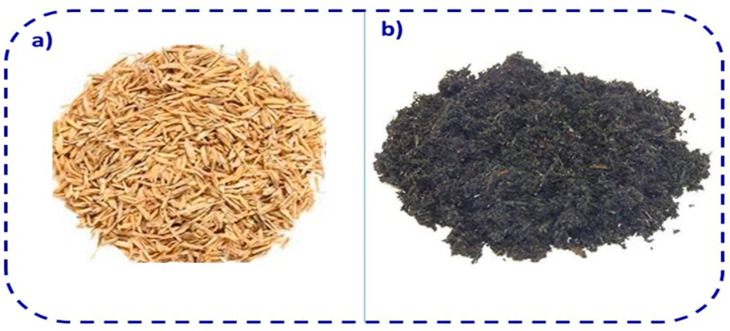
Photographic images of rice husk (**a**) and rice husk ash (**b**).

Rice husk ash (RHA) is one of the highly reactive supplementary cementitious materials for the construction of concrete structures [84,85]. The photographic image and chemical composition of RHA are given in Figure 6 and Table 4, respectively. The replacement of Portland cement by RHA not only improves the durability of concrete but also minimizes the production of cement and reduces environmental pollution by storing large amounts of waste material [86,87]. Rice husk has been used as a boiler fuel in several industries, and the burning of rice husk produces a great deal of ash, which is known as RHA. It contains a large amount of amorphous silica particles, which have a high surface area [84,88,89,90,91]. Hence, it reacts with cement matrix to forms a CSH (calcium silicate hydrate) gel, and reduces calcium hydroxide content because of the secondary hydration reaction caused by the RHA particles [92,93,94]. Subsequently, the strength of concrete can be greatly improved and the porosity reduced, which results in decreased cracking of concrete structures. Al-Khalaf and Yousif reported that the pozzolanic activity of RHA depends on the degree of grinding and the burning temperature. They reported that 40% replacement with RHA is suitable for concrete production without affecting the traditional concrete strength [95]. The uncontrolled burning of rice husk can adversely affect the amorphous nature of silica and can produce crystalline microstructures of silica [96,97]. Hence, the burning temperature of RHA is recommended to be 500–700°C. This temperature is suitable for the production of a large volume of amorphous silica with a high surface area [98,99]. However, few researchers have studied whether the partial replacement of Portland cement by RHA in concrete yields good strength without special processing of the RHA [100,101].

Mechanical grinding is essential to achieve the necessary fineness and high surface area of the RHA particles. Corderiro et al. studied the suitable grinding time of RHA and reported that a minimum of 120 min of grinding time was sufficient to produce a fine particle with a high surface area which increases the pozzolanic activity index [103]. Song et al. found that reduced chloride permeability and porosity of concrete were achieved by replacing RHA up to 30% in cement without compromising mechanical and corrosion resistance properties [85]. The use of RHA in cement replacement usually decreases the workability and increases the water demand. Kishore et al. reported that 10% RHA is the optimum replacement level and gives improved workability and strength [104]. However, some other researchers demonstrated that 30% replacement of cement by RHA involves no significant change in the strength, workability and permeability properties [105]. Freeze-thaw resistance of concrete using 15% of RHA exhibited good freeze-thaw efficiency and was be able to withstand 300 cycles [106]. However, Park et al. reported that the freeze-thaw resistance of concrete using RHA is slightly less when compared to silica fume-admixed concrete at up to 300 freeze-thaw cycles. They also reported that when freeze-thaw cycles are extended up to 600 cycles, the freezing-edge resistance of concrete using RHA was equal to that of concrete made with silica fume [107]. RHA-containing concrete is resistant to freeze-thaw cycles due to the microporous structure of RHA, which helps to expand water during freezing, thus reducing the growth of internal pressure [108,109]. RHA effectively reduces the sulphate deterioration of concrete. RHA contains silica, which chemically binds with free calcium hydroxide in cementitious compounds, rendering it unavailable for the sulphate reactions which reduce concrete permeability. This also reduces the amount of reactive aluminates available for sulphate reaction. Chatveera and Lertwattanaruk reported that a replacement level of 30% of RHA-admixed cement mortar showed less expansion when exposed to a high-sulphate environment; however, the expansion of cement mortar increased when increasing the replacement level of RHA, and also reduced the traditional concrete strength [110]. RHA-blended concrete improved the strength, porosity, and corrosion resistance of concrete as well as resistance to sulphate attack [92,102]. All these results prove beyond doubt that RHA is an effective SCM; at an optimal replacement level up to 30% of Portland cement, RHA improves strength and reduces sulphate reactions, chloride diffusion, and the corrosion rate of embedded steel rebar.

#### Pozzolanic Reactions of RHA

The pozzolanic reaction is favorable in RHA when reactive silica is in an amorphous state. In general, the amorphous silica that is ubiquitous in pozzolanic material reacts strongly with lime compared to the material in the crystalline phase. The pozzolanic reaction mechanism is an acid–base reaction between silicium acid (H_2_SiO_4_^2−^) from the reactive amorphous silica and calcium hydroxide (Ca(OH)_2_). The pozzolanic reaction mechanism are given below.

Conventional cement hydration reactions:

(2) $$\mathrm{Cement}\left(C_{3}S\right)+{\;H}_{2}O\;\to {\;\mathrm{Ca}}^{2+}+H_{2}{\mathrm{SiO}}_{4}^{2-}+{\;\mathrm{OH}}^{-}$$

(3) $${\mathrm{Ca}}^{2+}+H_{2}{\mathrm{SiO}}_{4}^{2-}\;\to C-S-H+\mathrm{Ca}{\left(\mathrm{OH}\right)}_{2}\;\left(\mathrm{Remaining}\;\mathrm{Calcium}\;\mathrm{hydroxide}\right)$$

Cement hydration reaction with addition of pozzolanic materials:

(4) $$RHA\;\left(SiO_{2}\right)+H_{2}O\;\to \;H_{2}SiO_{4}^{2-}\;$$

(5) $$Ca{\left(OH\right)}_{2}\;\left(Remainig\;Calcium\;hydroxide\right)+H_{2}O\;\to \;Ca^{2+}+{\left(OH\right)}^{-}\;$$

(6) $$Ca^{2+}+H_{2}SiO_{4}^{2-}\;\to C-S-H\;\left(Secondary\;CSH\;formation\right)\;$$

**Figure 7:**
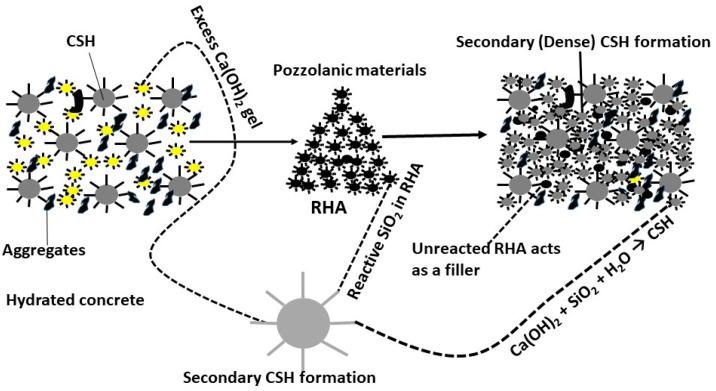
Hydration process for cement with the addition of RHA.

From the above reactions, it can be understood that the formation of additional C-S-H gel can improve the strength and durability of concrete. It is a fact that the small particle size of RHA enables it to permit Ca^2+^ ions to diffuse internally, thus allowing the hydration of cement and pozzolanic reactions to continue for a longer period of time. The hydration process of cement with the addition of RHA is depicted in Figure 7.

### 2.5. Sugarcane Bagasse Ash (SBA)

**Figure 8:**
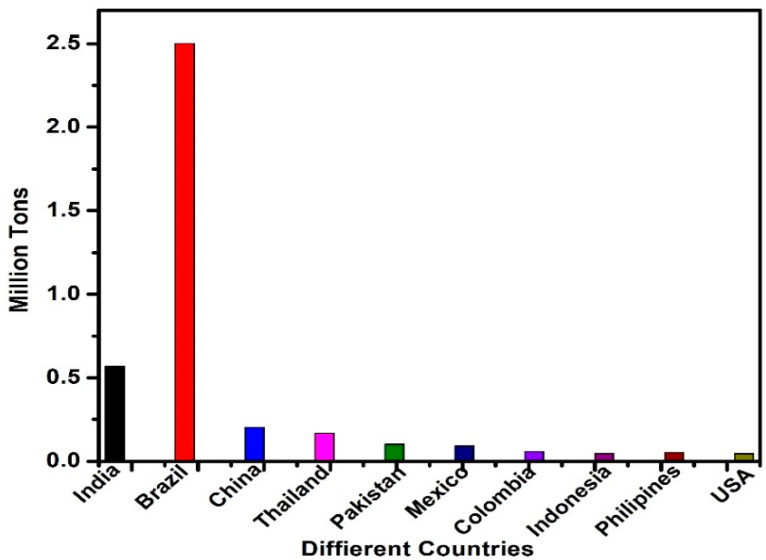
Worldwide production of SBA and source; data from [114].

Sugarcane bagasse ash (SBA) is a major residue of the sugar industry. Bagasse is an agro-waste material that remains after the extraction of sugarcane juice in the sugar industry. After extraction, fibrous sugarcane (bagasse) is partly used as the main fuel in the sugar industry for power generation [111,112]. The remaining material after burning bagasse is called SBA, which is considered a waste material. The sugar industry produces approximately 72.6 million tons of SBA, which is stored in landfills [113] and causes environmental problems. The safety and environmental concerns provide continuous motivation to the research community to utilize bagasse ash in the construction industry, where it is used as a secondary pozzolanic material for constructing concrete structures. The worldwide production of SBA is given in Figure 8 [114] and its chemical composition is given in Table 5.

Studies have revealed that the bagasse ash contains 63–70% silica [113,115,116,117,118,119]; however, a few recent studies have also indicated that bagasse ash contains more than 76–80% SiO_2_ [120,121,122], as well as other oxides, namely Al_2_O_3_ and Fe_2_O_3_. SiO_2_, Al_2_O_3_ and Fe_2_O_3_ are pozzolanic substances, and when is mixed with cement act as a cementitious material [123]. Bahurudeen and Santhanam et al., [115] reported that the obtained bagasse ash cannot be directly used in construction, as it requires grinding and thermal treatment to improve the fineness and remove impurities. Nevertheless, some recent studies have reported that bagasse ash does not require any heat treatment. Sometimes grinding may be recommended to improve the fineness [123]. It has been reported that the addition of bagasse ash to concrete enhances the workability of the cementitious mixer, i.e., when the addition of bagasse ash to concrete was increased (5–25%), the compaction factor also increased, which refers to improved workability [116,124,125]. Hussein et al. reported that 30% replacement level of cement with SBA increases its slump [117]. Singh et al. studied the hydration effect of bagasse ash with cement and observed better performance even at a 10% replacement level [118]. Bagasse ash has been found to improve the mechanical properties of concrete, including consistency, setting time, workability, compressive strength and permeability [126].

It has been reported that M20 concrete made using 5–25% of SBA showed greater compressive strength even at a 5% replacement level when compared to control concrete [116,127]. The bagasse was subjected to thermal treatment at 600 °C for 1 h, and M25 grade concrete was made with various replacement levels up to 30% of cement by SBA [127,128,129]. In this study, it was observed that the concrete containing 20% of SBA showed better compressive strength than reference concrete. Dhengare et al. examined the effect of SBA in M25 and M35 grade concretes and reported that the maximum split tensile strength was observed at 15% replacement level [130]. Moreover, the concrete containing SBA reduced the chloride permeability of the concrete. Hussein et al. and Andrade established a 20% replacement of Portland cement by SBA, which effectively increased its resistance to chloride diffusion without affecting the other properties of the concrete [126,131]. Ganesan et al. examined the chloride diffusion coefficient of SBA-blended M25 concrete with various replacement levels (0–30%) in Portland cement at two different curing ages, namely 28 and 90 days [126]. A significant reduction in chloride diffusion was noticed at the 25% replacement level, however, it started to increase the chloride diffusion at the 30% replacement level in both curing periods. Shafiq et al. reported that chloride diffusion was decreased at up to 25% SBA level, and was thereafter increased at 30% SBA level [132]. This increase may be due to the unreacted portion of SBA content in the concrete, which leads to an increase in porosity and thereby increases chloride penetration into the concrete. Rukzon et al. reported that the replacement of cement with SBA significantly decreased the corrosion rate of steel in concrete structures [119,133]. Ganesan et al. investigated the corrosion-resistant properties of SBA-blended concrete with various replacement levels and found that 10% SBA-blended concrete improved corrosion resistance properties without hampering the other properties of the concrete [134]. However, this depends on the type of embedded reinforcing steel in concrete. Ramakrishnan et al. evaluated the porosity and water absorption of blended concrete containing up to a 20% SBA replacement level [135]. They concluded that the porosity and water absorption of concrete decreased with an increase in the percentage of SBA up to 15%. The same authors also examined the acid resistance properties of SBA-containing concrete, with similar results to the water absorption and porosity test. Rambabu et al. attempted to study the acid resistance behavior of 0–20% SBA blended concrete in acidic media such as H_2_SO_4_ and HCl (1% to 5%). They concluded that acid resistance with up to 10% SBA replacement level was better than control concrete [136]. Singh et al. investigated the acid resistance properties of cement with SBA (10%, 20% and 30%) exposed to N/60 H_2_SO_4_. They found improved acid resistance properties in the blended cement when compared to the control mix at a 10% replacement level [118]. Gupta et al. made an investigation of the sulphate resistance of (0–10%) SBA blended concrete exposed to 1%, 3% and 5% of Na_2_SO_4_ solution. They concluded that 10% of SBA blended concrete reduces the sulphate attack when compared to control concrete [137]. Based on the above literature, it was confirmed that SBA is said to be a pozzolanic material and able to reduce free Ca(OH)_2_ in a cement matrix, which is owing to the pozzolanic reaction. The optimum replacement level of cement is 10% SBA, which improves strength and durability, decreases the acid attack, chloride diffusion and corrosion rate of embedded steel rebar.

#### Influence of SBA in Cement Matrix

SBA contains 77–88% silica, which is favorable for pozzolanic reaction due to the availability of amorphous silica. The presence of amorphous silica in SBA reacts with free Ca(OH)_2_ in cement matrix, which produces the secondary C-S-H gel and improves the compressive strength of concrete. Moreover, SBA contains Al_2_O_3_, which reacts with Ca(OH)_2_ and leads to the formation of C-A-H, which can resist sulphate attack.

(7) $$\mathrm{Ca}{\left(\mathrm{OH}\right)}_{2}+\mathrm{SBA}\;\left({\mathrm{Al}}_{2}O_{3}\right)\;\to C_{2}\mathrm{AS}+C_{3}\mathrm{AS}$$

Furthermore, the unreacted silica in SBA acts as a pore filler, which can reduce porosity and voids in the concrete. It is also helpful for resistance to chloride penetration, and reduces the corrosion rate of steel rebar. Increasing the addition of SBA by more than 10% increases the water demand and reduces workability and compressive strength, due to the fineness and surface area of SBA.

### 2.6. Tire-Derived Fuel Ash (TDFA)

As the automobile industry has grown exponentially worldwide, the usage of tires has continuously increased. Some 1.6 billion new tires are manufactured every year, generating around one billion used tires which require proper disposal [138]. Tires do not break down naturally, as they are made of non-biodegradable materials [139,140]. In recent years, the disposal of waste tires has become a major environmental concern. Waste tires mixed with coal or wood are used as fuel in the paper industry, cement kilns, and power plants. The residue of burned waste tires collected from the boilers is known as tire-derived fuel ash (TDFA). The use of waste tires as fuel is increasing; thus, the amount of TDFA production has also increased, which causes environmental problems if it is stored in landfills. These safety and environmental concerns have provided continuous motivation to the research community to investigate the utilisation of TDFA in the construction industry, where it is used as a substitute for conventional fillers in asphalt concrete [141]. The chemical composition of TDFA is given in Table 6 [142]. TDFA contains 27.5–31.1% SiO_2_, Al_2_O_3_, and CaO, and these oxides are suitable for pozzolanic materials. According to the ASTM C-618-08a, TDFA is considered as a class C pozzolana because the sum of the ratio of oxides is more than 50% [10]. TDFA has a lower SiO_2_ content compared to other ashes like fly ash, RHA, and SBA. Al-Akhras and Smadi investigated the effects of TDFA as a sand replacement in cement mortar. In their study, 2.5%, 5% 7.5%, and 10% TDFA replaced sand weight [143]. The authors reported that the workability of the mortar was reduced with increasing percentage of TDFA, which is due to the higher surface area and lower density of TDFA compared to sand. Because TDFA can absorb more water, and reduces workability, proper compaction is required for the preparation of mortar. Hyeok-Jung et al. used water reducing agents to increase the workability of concrete [142]. Al-Akhras and Smadi have reported that the air content of TDFA concrete is reduced with increasing percentage of TDFA [143]. In addition, the final setting time of mortar increases with increasing amounts of TDFA. The same authors showed that the compressive and flexural strength of mortar increases continuously with an increase in TDFA percentage up to 10%. Furthermore, 10% TDFA mortar showed better resistance to chloride diffusion and freeze-thaw damage than control mortar. Hyeok-Jung et al. investigated the durability of concrete with TDFA [142] by casting concrete using TDFA as a fly ash replacement. The concrete containing cement with 20% FA was substituted with 3.0–12% TDFA. The study showed that the 28-days compressive strength with 3–12% TDFA had no significant effect on the strength development of the concrete. However, the 90-day compressive strength of the concrete increased continuously with an increase of 6%, 9%, and 12% in TDFA, which was due to the long-term strength development of fly ash. Based on their experimental results, they concluded that concrete containing 6.0% TDFA has improved strength development and reduced porosity, carbonation, and chloride diffusion. Choi et al. also examined TDFA as an alternative filler for asphalt mixture [141].

Other research has investigated the use of crumbed and shredded waste tires in fine aggregate and coarse aggregate [144,145]. Kardos and Durham used scraped rubber from waste tires as a replacement for sand at ratios of 0%, 10%, 20%, 30%, 40%, and 50% in their study, to determine any volatile organics leached from the crumb rubber-containing concrete. They reported that 30% replacement of sand by crumb rubber is optimal for the production of concrete pavement [146]. Arulrajah et al. investigated recycled aggregates blended with tire-derived aggregate (TDA) at a ratio of 1–3%. In their study, they found that recycled aggregates blended with 3% tire-derived aggregates were suitable for pavement subbase application [147]. However, few research data are available on the incorporation of TDFA or TDA in concrete construction. Meanwhile, further research is required in order to determine the optimal mix conditions as well as the influence of SO_3_ content in concrete, as higher SO_3_ contents are present in TDFA.

#### Pozzolanic Action of TDFA

TDFA contains 25–31% SiO_2_, 24–36% CaO, 4–14.6% Al_2_O_3_ and a small amount of carbon material (Ref Table 6), which can chemically react with the calcium hydroxide in the cement matrix and favors the pozzolanic reaction in TDFA admixed concrete. The small amount of carbon material in TDFA increases the formation of Ca(OH)_2_ phases [142]. Moreover, the presence of CaO and SiO_2_ in TDFA reacts with water during concrete mixing, undergoing the following reaction:

(8) $$\mathrm{TDFA}\;\left(\mathrm{CaO}\right)+2H_{2}O\;\to \mathrm{Ca}{\left(\mathrm{OH}\right)}_{2}$$

(9) $$\mathrm{TDFA}\;\left({\mathrm{SiO}}_{2}\right)+{\;H}_{2}O\;\to {\;H}_{2}{\mathrm{SiO}}_{4}^{2-}$$

## 3. Chemical Additives/Admixtures

**Figure 9:**
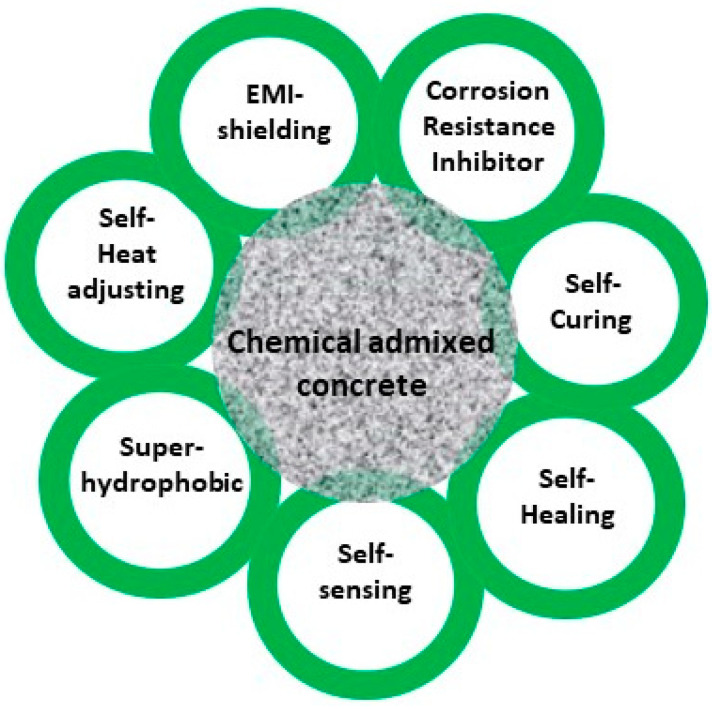
Various applications of chemical admixtures used in concrete.

Chemical admixtures are ingredients in concrete other than Portland cement, water, and aggregates that are added to concrete in very small amounts immediately before or during mixing. The chemical admixtures are added to concrete for some specific functions, including as air entrainers, water reducers, set retarders or accelerators, superplasticizers and some special admixtures for such purposes as corrosion inhibition, self-curing, self-healing, electromagnetic shielding, self-temperature adjusting and hydrophobic properties. In this review, special emphasis is given to chemical admixtures for corrosion inhibition, self-curing, self-healing, electromagnetic shielding and self-temperature adjusting and hydrophobic materials and their applications in concrete. The various applications of chemical admixtures used in concrete is given schematically in Figure 9.

### 3.1. Corrosion Inhibiting Admixtures

**Figure 10:**
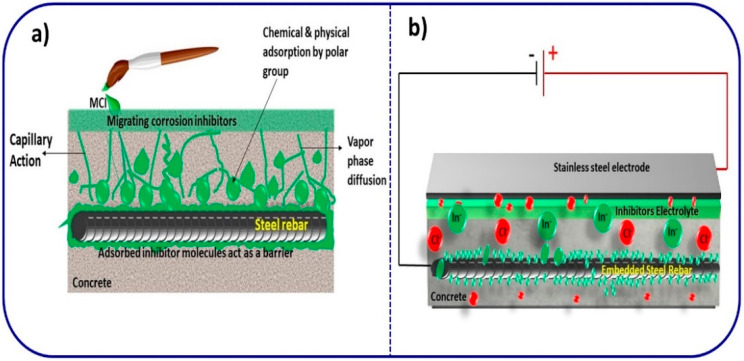
Schematic diagram of the migrating (**a**) and electro-injection (**b**) methods of corrosion inhibition.

Corrosion is a primary concern for the durability of concrete structures. The presence of aggressive ions in the atmosphere affects concrete structures and reduces the durability of concrete [148]. Corrosion-inhibiting admixtures represent one of the best ways to protect the embedded rebar from corrosion and prolong the lifetime of concrete structures [149]. Cement contains mostly inorganic ingredients; therefore, in the early days, inorganic-based corrosion inhibitors were widely used in the construction industries. Due to their high cost and non-availability, however, second-generation admixtures are instead based on organic, natural and green corrosion inhibitors. Different types of corrosion-inhibiting admixtures, namely admixed, migrating and electro-injection admixtures, are highly useful for protecting the steel reinforcement in concrete from corrosion. The mechanism of action of each type is different. For example, admixed-type inhibitors are directly added to the concrete during casting, and are thus considered as static; these passivate the steel. On the other hand, migrating-type inhibitors are applied on the surface of the concrete (Figure 10a) and are thus considered as dynamic, moving towards the steel rebar to offer corrosion protection [150]. In contrast, electro-injection (Figure 10b) formulations consist of inhibitors, passivators and transporting agents, which offer a high degree of corrosion protection even in chloride contaminated concrete [151]. During this process, a considerable amount of free chloride ions is removed from the concrete, which can present a practical difficulty and faces several problems in the field when using traditional inhibitors, i.e., toxicity, dangerous effects, high dosage levels and effects on the mechanical properties of concrete [152,153]. Hence, the need for environmentally friendly corrosion inhibitors to prevent corrosion problems in new and existing concrete structures has been studied [154]. However, a corrosion inhibitor does not stop the corrosion of metal, which prolongs the initiation of corrosion and reduces the rate of corrosion reactions between the metal and aggressive ions [155,156]. The inhibitors are distinguished on the basis of their chemical components, namely as organic and inorganic and (by the presence of a polar group) as anodic, cathodic or mixed (i.e., both positive and negative polar groups) [157]. Anodic inhibitors reduce the anodic reaction and form an insoluble passive film on steel rebar surfaces [158]. The most common anodic inhibitors are alkali metal nitrite and nitrate salts like calcium nitrite, sodium nitrite, potassium nitrite, molybdates, chromates, and orthophosphates [159,160]. For example, calcium nitrite resists corrosion by slowing down anodic reactions on the surface of steel rebar, as per Equation (10) [161].

(10) $$\mathrm{Fe}{\left(\mathrm{OH}\right)}_{2}\;\left(s\right)+{\;\mathrm{NO}}_{2}^{-}+H_{2}O\;↔\;\mathrm{Fe}{\left(\mathrm{OH}\right)}_{3}\left(s\right)+\mathrm{NO}+{\;\mathrm{OH}}^{-}$$

Neville has reported that sodium nitrites and calcium nitrites effectively protect against corrosion in the presence of chloride ions [162]. Das and Pradhan investigated the corrosion mitigation properties of concrete with a dosage of 3% NaNO_2_ by weight of cement [163]. These concretes were exposed to chloride and sulphate solution and based on the resulting experimental data, they reported that the addition of sodium nitrite decreased chloride diffusion in the concrete which, due to the formation of nitrite films, blocks the pores of concrete. The cathodic inhibitors suppress the cathodic reaction, so the reaction time of the corrosion rate was gradually decreased, increasing the durability of steel rebar. Cathodic inhibitors include phosphates, polyphosphate, zinc oxide, and magnesium oxide, silicates. Most cathodic inhibitors precipitate insoluble compounds on the cathodic region in the form of a protective layer [164,165,166,167,168,169,170]. The mixed inhibitors are another type of corrosion inhibitor; these suppress both anodic and cathodic reactions on the entire surface of steel rebar via adsorption, forming a hydrophobic passive film and thereby prolonging the rate of corrosion reactions on the rebar [171]. Mixed inhibitors consist mostly of amines and amino alcohol-based salts [172,173]. Bellal et al. have synthesized and studied 4-(3-Hydroxy-naphthalene-2-ylimino)-pentan-2-one (L2), a new mixed inhibitor with an inhibitor efficiency of 93% [174]. Meanwhile, organic inhibitors have become popular corrosion inhibitors in the construction industry; they are admixed in reinforced concrete to enhance the corrosion resistance of steel rebar [175,176]. Organic inhibitors can be adsorbed on metal surfaces through chemisorption [171]. Organic amines and alkanol amine-based salts are used as admixed inhibitors in concrete [177,178,179]. These corrosion inhibitors show dual action in concrete, which reduces both anodic and cathodic corrosion reactions [180]. It has been reported that the amines and alkanol amine and their salts reduce the corrosion reaction in carbonated concrete; however, in chloride-contaminated concrete they have a poor inhibition effect [181]. Ormellese et al. investigated the effect of the corrosion inhibition properties of amines/alkanol amines, amino acids and polycarboxylates. They reported that polycarboxylate showed better corrosion resistance compared to amines/alkanol amines and amino acids [182].

Brown et al. [183] and Heren and Olmez [184] have reported that increasing the concentration of amines in concrete reduces its compressive strength. El-Jazairi et al. studied the corrosion resistance and mechanical properties of cement mortars with sodium nitrite, potassium chromate, calcium nitrate and sodium benzoate [185]. Furthermore, several authors have investigated the effect of sodium nitrate and calcium nitrate in admixed concrete, which is an effective corrosion inhibitor [162,163,186,187]. However, the concentration of calcium nitrates increases the setting time and reduces the compressive strength of concrete; when utilized in low concentrations, the corrosion rate is also increased [188,189,190]. Hence, sodium and nitrite-based inhibitors require careful consideration in order to determine the essential amount; if it is too high or too low, it will affect both the concrete and the steel. Furthermore, Thangavel et al. studied 5% Al_2_O_3_ admixed cement mortar, which showed better corrosion resistance and improved compressive strength [191]. Muralidharan et al. investigated ordinary Portland cement (OPC), OPC + Fly ash at a 3:1 ratio, and Portland pozzolana cement (PPC) with composite inhibitive agents such as hydroxide, citrate and stannate. They concluded that the addition of composite inhibitors decreased the corrosion rate without affecting the traditional concrete compressive strength [192,193]. Saraswathy et al. examined the same composite corrosion inhibitors, admixed in OPC concrete under macrocell corrosion conditions [194]. Tungstate, phosphate, and sodium molybdate showed effective corrosion inhibition and re-passivated the pitting corrosion [195]. Song and Saraswathy investigated the influence of anodic, cathodic and mixed inhibitors in concrete under various conditions [157,179]. Based on the experimental results, they established that the mixed inhibitors improved the corrosion resistance and compressive strength of concrete.

Bastidas et al. examined the influence of three soluble phosphates, sodium monoflurophosphate (NaPO_3_F), disodium hydrogen phosphate (Na_2_HPO_4_) and trisodium phosphate (Na_3_PO_4_) and evaluated them in OPC paste and OPC mortar with steel rebar specimens [196,197]. They reported that these inhibitors showed better performance as migrating corrosion inhibitors. However, when admixed in concrete specimens, Na_2_HPO_4_ showed improved corrosion resistance properties and lower i_corr_ values. Table 7 shows the literature review of the most widely used inorganic and organic corrosion inhibitors in concrete and in simulated concrete solution [198,199,200,201,202,203,204,205,206]. From Table 7, it can be observed that the inhibitors are generally focused on simulated concrete with and without chloride medium, while a few studies have been conducted on cement mortar and concrete. The anodic, cathodic and mixed/organic inhibitors protect steel from chloride-induced in of concrete structures; however, there are no conclusions indicating that the mechanism of corrosion inhibition can decrease the corrosion rate for long-term applications. Hence, more extensive examination of such aspects of these inhibitors as chloride content and environmental friendliness in real concrete structures, without affecting the cement setting time and strength properties, etc., is required [155,160].

### 3.2. Self-Curing Concrete

**Figure 11:**
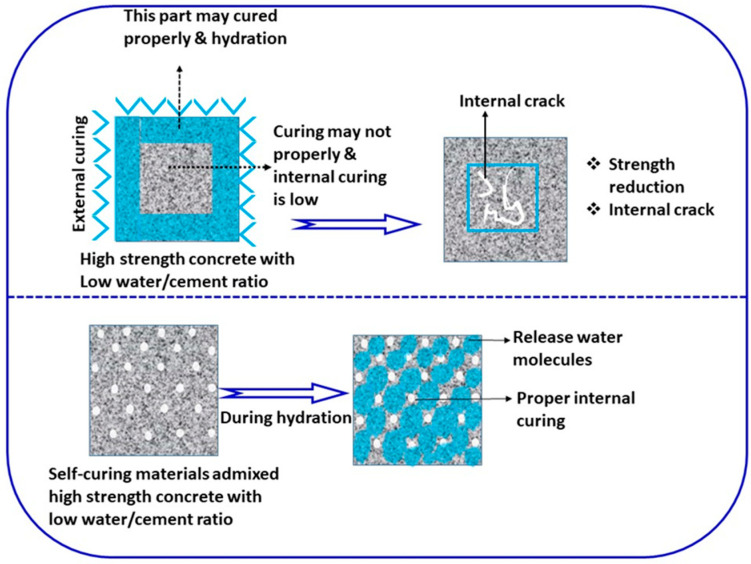
Illustration of self-curing mechanism in concrete.

At present, high-performance or high-strength concretes are widely used in most industrial applications. High strength concrete is designed using a low water:cement ratio, and the strength and durability of concrete can be further increased by proper external water curing. During external curing, a large amount of water is applied to the concrete surface; however, the internal concrete surface area may not be properly cured by traditional external curing. This can lead to the hydration reaction not being completed, and to loss of strength and the formation of microcracks in concrete structures. Insufficient rain and scarcity of water is a critical problem in arid and semi-arid regions; self/internal curing is a potential solution to overcome this problem [207]. The schematic diagram of the self-curing mechanism is given in Figure 11. Recently, water-containing or super-absorbing additives have been added to concrete mix during casting, through which internal curing can be achieved and the process of microcrack formation reduced [208]. Polyethylene glycol, sodium polyacrylate, polypropylene glycol, polyvinyl alcohol, and polyacrylamide are generally used for making self-curing concrete. Friedemann et al. have reported that carboxylates and sulfates of polysaccharide-based super-absorbing polymer performed better as internal curing agents [209]. Hu et al. developed a Ca^2+^ based ion-responsive superabsorbent hydrogel to achieve concrete self-curing and self-healing as well as increased compressive strength in concrete structures [210]. It has been reported that polyethylene glycol admixed in concrete with different concentrations can improve physical properties and water maintenance. Polyethylene glycol contains OH- groups, which can absorb water during the casting of the concrete and return the water during cement hydration reactions [211,212,213]. Teja et al. studied the mechanical properties of self-curing concrete containing 1% and 2% of polyethylene glycol with 5% of calcinated zeolite [214]. In their study, they concluded that calcinated zeolite with polyethylene glycol showed better compressive strength than control concrete. Self-curing concrete consists of various supports which reduce cracks and shrinkage, resulting in improved durability of concrete. The most advantageous aspect of the self-curing process is that it reduces labour requirements during the curing process [215]. However, more extensive examination is still required to making truly eco-friendly concrete structures.

### 3.3. Self-Healing Concrete

**Figure 12:**
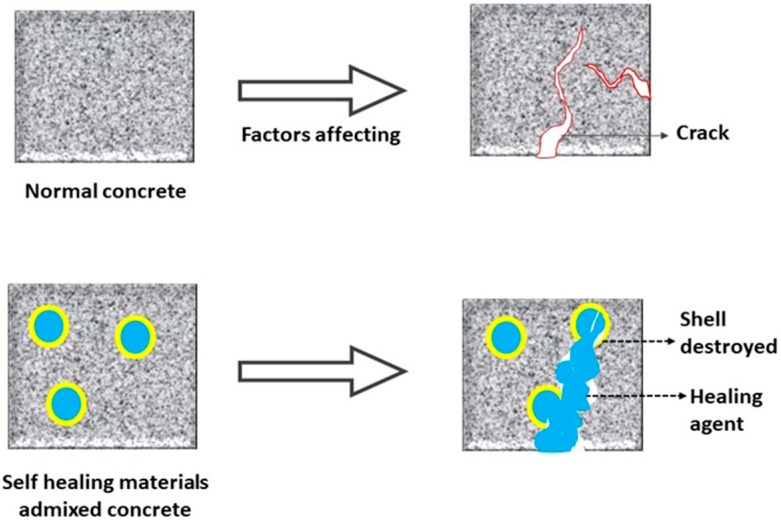
Illustration of the self-healing mechanism in concrete.

Concrete is one of the most generally used construction materials worldwide, but it is easily susceptible to cracking [216,217,218], which is due to chemical and dry shrinkage in concrete [219]. The formation of cracks shortens the durability of concrete because they offer an easier footpath for the penetration of aggressive ions into the concrete [220]. Hence, introducing self-healing materials into concrete may increase its durability [221,222,223]. Self-healing materials are also known as self-repairing materials, and generally result in concrete capable of automatically repairing cracks without any external inspection or human involvement. A schematic diagram of the self-healing concrete mechanism is given in Figure 12. Self-healing concretes can be separated into autogenous and autonomous healing types. Autogenous healing occurs by two methods, hydration and carbonation [224]. In the hydration healing method, water molecules propagate through the cracks and react with the unhydrated Ca^2+^ on the cracks to heal narrow cracks [225]. In the carbonation healing process, the unhydrated cement particles (Ca(OH)_2_) react with CO_2_ to form CaCO_3_, healing cracks in the concrete [226]. On the other hand, in autonomous healing, many different approaches have been studied, i.e., the micro-capsule method, vascular method, electrodeposition method, and microbial method. However, autonomous healing requires a trigger in order to activate the process [227,228]. For example, a microcapsule is generally triggered by crack occurrence. It has been shown that urea-formaldehyde microcapsules filled with epoxy resin and gelatin microcapsules filled with acrylic resin result in good self-healing properties of concrete under compressive conditions [229]. This is because cracks form in the concrete under loading conditions, which breaks down the capsules and releases the epoxy and acrylic resins needed to cure the cracks. Wang et al. studied the performance of microcapsule-based self-healing properties of concrete under laboratory and field conditions [230]. In their study, they used urea–formaldehyde resin as the shell and epoxy resin as the healing agent. They concluded that the addition of microcapsules to concrete resulted in better chloride diffusion resistance; however, the microstructure of the concrete specimen showed both positive and negative effects from the addition of microcapsules. Al-Tabbaa et al. studied the performance of microcapsule-based self-healing concrete in field application in the UK [231]. In their study, micro-capsulated sodium silicate was used for the casting of self-healing concrete. They concluded that the microcapsule-based concrete showed improvement in the reduction of crack width, crack depth, and recovering permeability of concrete structures.

Du et al. investigated the application of paraffin microcapsules with toluene di-isocyanate for self-healing concrete [232]. They reported that the optimal content of 3% microcapsule in cement mortar showed better self-healing capacity than the control mortar. Further, Sun et al. investigated the fatigue behavior of asphalt concrete with microcapsule-induced self-healing properties [233]. In their study, they used melamine–urea–formaldehyde microcapsules as a rejuvenator. Based on the results, the asphalt concrete with 3% microcapsules played an essential role in improving the self-healing ability of the asphalt mixture. Dong et al. studied novel chemical self-healing microcapsules for corrosion mitigation of rebar in concrete [234]. In their study, they used ethyl cellulose (EC) as the microcapsule shell, and both NaNO_2_ and monofluorophosphate as healing materials. They used the X-ray micro-computed tomography (XCT) technique to monitor the corrosion status of rebar in concrete. Based on the experimental results, they reported that the two kinds of self-healing systems showed better performance in healed the cracks caused by corrosion. However, autogenous and autonomous healing is limited to small cracks and was not suitable for wider cracks, and its effectiveness depends on the water content [235]. 

Another method involves the utilization of bacteria in self-healing concrete, and can be applied to external cracks by spraying or injection into concrete [236]. Ureolytic bacteria are commonly used in self-healing concrete structures, and are an environmentally friendly method of repairing cracks in concrete. These bacteria convert urea into ammonium and carbonate, thus producing CaCO_3_ in cracks [237,238,239,240]. These precipitated crystals of CaCO_3_ are able to heal cracks in concrete. This bacteria-based self-healing is very effective, and able to repair wider cracks [238]. However, the use of bacteria is very challenging in the alkaline environment of concrete [241]. As a result, autogenous, autonomous and bacteria-based self-healing concrete technology is a double-edged sword [242]. There is a need for more extensive investigation in future real concrete structure application by considering such various aspects as the particle size of microcapsules and the enhancement of bacterial growth by providing required nutrients [230,242].

### 3.4. Super-Hydrophobic Concrete

**Figure 13:**
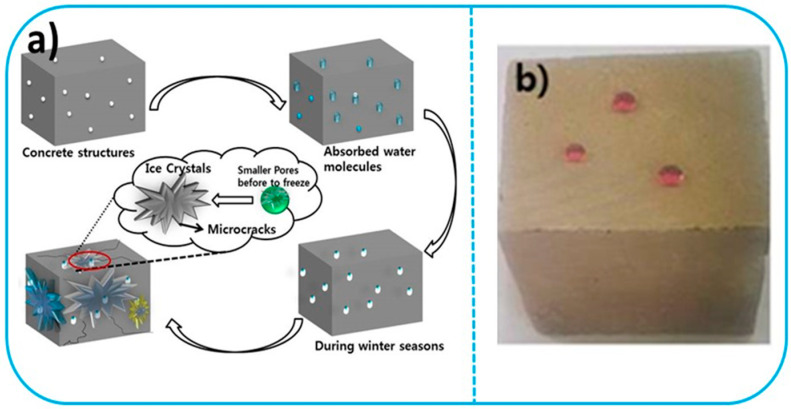
Schematic diagram of the mechanism of crack formation in concrete (**a**); Incorporation of hydrophobic materials admixed in concrete (**b**) [255].

Reinforced concretes can be vulnerable to cracking depending on the environmental conditions [216,217,218]. Because concrete structures are naturally porous and hydrophilic, they absorb water along with some aggressive ions via their micropores [243]. The absorbed water molecules become frozen in extreme cold conditions, and consequently the internal stress on the concrete increases, which creates microcracks on the surface [244,245]. As a result, the durability is reduced, with potentially catastrophic effects. The schematic diagram of the mechanism of crack formation in concrete is given in Figure 13a. Incorporating hydrophobic properties in concrete can prevent the absorption of water (shown in Figure 13b), which can help to reduce the formation of cracks during winter seasons [246,247,248,249]. However, the hydrophobic mechanism of concrete is not completely similar to other materials. Thus far, several authors have established the hydrophobic properties of some chemical-admixed methods. The biomimetic superhydrophobic properties of concrete occur by two methods, namely coating [250,251] and admixture using hydrophobic materials [252]. Biomimetic superhydrophobic preparation using coating techniques has shown better water resistance properties, however, the coating is easily peeled off by external mechanical forces and also weakens the adhesion between the coating and the concrete surface [253,254]. 

Superhydrophobic materials are admixed in the concrete during casting as a water resistant material. A biomimetic superhydrophobic surface can be developed using silane and silicone hydrophobic materials. For example, Song et al. investigated the influence of fluoroalkylsilane with filler materials admixed in concrete [253]. In their study, they reported that the concrete surface had a very high surface roughness and superhydrophobicity, with contact angles of 158 ± 0.8°. Karthick et al. investigated the influence of 1H,1H,1H,2H-perfluorodecyl-triethexysilane enriched with nanomaterials admixed in concrete. This modified cement mortar showed excellent water resistance and superhydrophobic properties, with a contact angle of 162° [255]. Furthermore, Zhu et al. [256] and Xue et al. [257] studied the integral hydrophobicity of concrete, which was improved by using octyltriethoxysilane-based materials. They reported that silane-based materials improved the water resistance properties and durability of concrete. However, silane and silicone are expensive, and cannot be used in large concrete structures. These materials also hindered the cement hydration reaction between cement and water, severely reducing the concrete strength [255]. Liu et al. prepared a hydrophobic polymer concrete with fly ash, using different types of polymers such as polyacrylic ester styrene-butadiene, rubber latex, and organic silicon as waterproofing agents [258]. The mechanical and water-permeable properties of the polymer-modified concrete were examined. In this study, they concluded that 1–2% polymer was suitable for concrete and improved the compressive and permeability properties of concrete. The organic silicon waterproofing agent used for the concrete exhibited better performance than the other two polymers. Other researchers have examined the influence of stearic acid admixed in concrete, as it is a low-cost hydrophobic material [259,260,261]. However, stearic acid does not dissolve in water, hence it is a difficult challenge to homogeneously mix it into concrete [262]. Feng et al. prepared concrete with a waterborne stearic acid emulsion to improve the internal hydrophobicity of the concrete mixture [262]. They concluded that stearic acid emulsion-modified cement mortar showed better internal hydrophobicity with a contact angle of 132°. The compressive and flexural strength of the modified cement mortar was slightly lower (16.2% & 20.0%) than the control cement mortar. A literature review of studies on super-hydrophobic concrete surface contact angles is given in Table 8 [261,263,264,265]. Superhydrophobic materials improve the surface roughness and internal hydrophobicity of concrete; however, further experimental investigation is required for long-term application without affecting the usual mechanical properties of concrete as it is still unknown for how long hydrophobic materials will decrease water permeability.

### 3.5. Electromagnetic (EM) Wave Shielding Concrete

**Figure 14:**
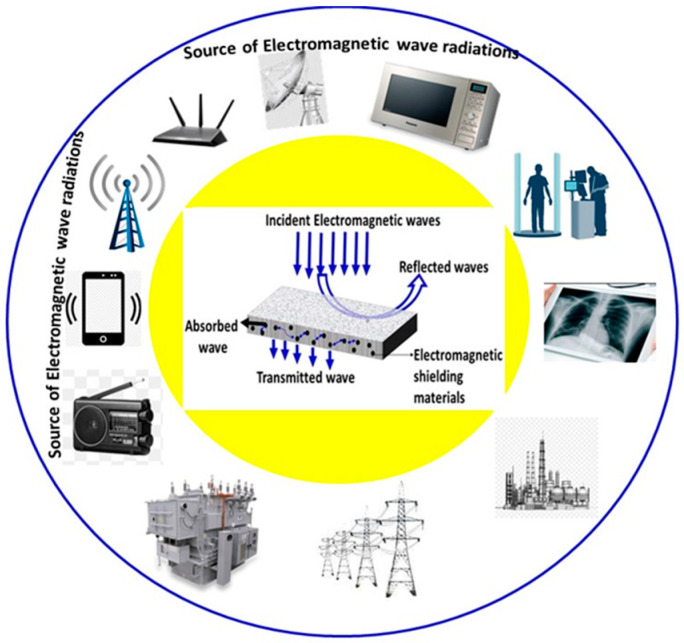
Schematic diagram of the electromagnetic source and shielding mechanism of concrete.

Wireless electronic devices are proliferating rapidly in the contemporary technological world, and the electromagnetic waves emitted from these devices are likewise increasing [266]. EM waves from many electronic devices (mobile phones, microwave ovens, etc.) have become a new form of pollution which can cause the malfunctioning of electronic devices, interfering with military and security telecommunications and even potentially affecting the health of humans and other livings beings [267,268]. To prevent this, electromagnetic wave interference (EMI) is essential in the contemporary world. The sources of electromagnetic waves and shielding mechanisms are illustrated in Figure 14. Generally, in the EMI reflection shielding method, the concrete should have mobile charge carrier materials, namely concrete mixed with some conducting filler materials such as carbon materials, conductive polymers and metal fibers [269,270,271]. The free electrons which exist in these materials interact with EMI; as a result, the concrete reflects EMI radiation [272,273]. Carbon-based materials (carbon fiber, carbon black, graphite powder) are most commonly used as filler materials for EMI reflection shielding concrete [274]. Zhang et al. studied the effects of graphite powder admixed in concrete and observed that the EMI shielding efficiency (SE) at 18.0% graphite volume level was 10–40 dB of SE in the range of 200–1600 MHz [275]. Carbon fiber was used by Nakamura and Shintani to prepare concrete with an SE of between 26 and 54 dB in the frequency range of 30 MHz–1 GHz [276].

Liu et al. examined the effect of helical CNT-coated carbon fiber composite, which exhibited reflectivity of 32 dB in the frequency range of 8.2 GHz to 18 GHz. It has been reported that an SE of 30 wt% graphene oxide can be obtained by incorporating ferrofluid in concrete, leading to an SE of 46 DB in the frequency range of 8.2–12.4 GHz [277]. Jung et al. examined the effect of CNT admixed in concrete and reported that the SE of 0.8% and 1.0% of carbon nanotube (CNT) admixed concretes was ~20 dB and ~45 dB in the frequency of 1 GHz, respectively [278]. Wen and Chung have reported that the SE of 0.72% with stainless steel fibers admixed in concrete was 70 dB at the frequency of 1 GHz [279]. 

In the EMI absorption shielding method, concrete is made using highly magnetic materials such as magnetite, hematite and other ferromagnetic materials [280,281,282,283,284]. These materials have magnetic properties which are able to provide magnetic dipoles, which interact with EMI and, as a result, can absorb EMI radiation [284]. These conductive materials and magnetic ferrites are appropriate materials for constructing EMI absorbing concretes. Lu et al. examined the effect of calcined TiO_2_ with clay admixed into the concrete and reported that TiO_2_-containing concrete showed good EMI absorption and excellent compressive strength [285]. Zhang and Sun investigated Mn-Zn ferrite admixed concrete and observed that the SE of 30% Mn-Zn ferrite concrete was 15 dB at the frequency range 11.4–18 GHz [283]. It has been reported that Ferro-boron admixed concrete showed better absorption of neutron and gamma radiation [286,287]. Micheli et al. studied the effects of multi-wall carbon nanotubes (MWCNT) admixed in concrete at various weight percentages (0%, 1%, and 3%), and observed that the SE of 3% MWCNT was 10–35 dB in the frequency range of 0.8–8 GHz [288]. Nano-Fe_3_O_4_ fluids were used by He et al. to prepare concrete with 5% Fe_3_O_4_, which had a better SE in the frequency range of 8–18 GHz [289]. Ogunsola et al. simulated the EMI-shielding properties of concrete with steel fibers and observed that the SE of concrete was 7–9 dB in the frequency range of 0–4 GHz [290]. EM shielding/absorbing concretes are very useful for preventing EM waves and suitable for good environmental adaptability. EMI-absorbing concrete is more convenient than EM-reflecting concrete because the reflected wave may interact with the incident wave [291,292]. The electromagnetic shielding effectiveness of concretes with different chemical admixtures are given in Table 9 [271,293,294,295,296,297,298,299,300,301,302,303,304,305]. It can be observed from Table 9 that carbon-based materials are the main focused of electromagnetic shielding properties in concrete. However, the main disadvantages of EMI-shielding concretes are reduction of compressive strength and that, while it can absorb significant bandwidth, it cannot absorb 100% [306]. Therefore, further research is needed to develop EMI-shielded concrete with new materials that are less expensive and have the appropriate shielding properties as well as good compressive strength.

### 3.6. Self-Temperature Adjusting Concrete

**Figure 15:**
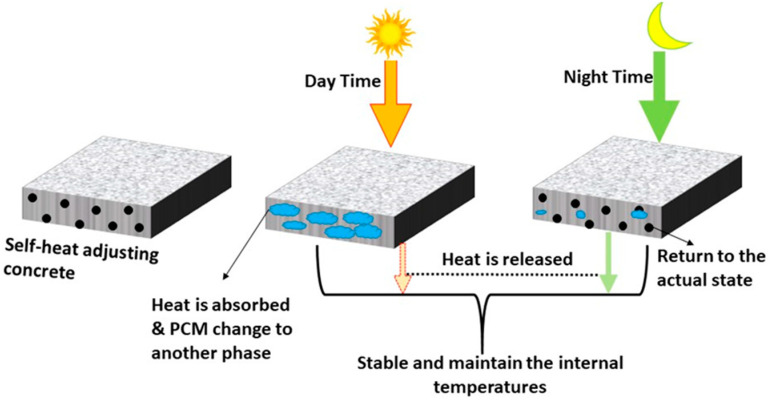
Schematic diagram of the mechanism of self-temperature adjusting concrete [310].

Indoor concrete buildings consume more energy for cooling during the summer seasons, which amounts to one-third of energy consumption in most countries [307]. Hence, it is very important to look for alternative effective methods to reduce energy usage. There have been several methods introduced in order to reduce the consumption of energy. For the past two decades, a great deal of research has been focused on the utilisation of phase change materials (PCMs) in concrete in order to mitigate thermal effects [308,309,310,311,312]. PCMs can maintain the temperature owing to their phase-changing transition. PCMs are able to absorb or release heat depending on the surrounding temperatures [313]. Depending on temperature, during the daytime PCMs absorb heat, shifting from solid phase to liquid phase, and then at night shift back to a solid phase and release heat into the surrounding environment [314]. A schematic diagram of self-temperature adjusting concrete is given in Figure 15. The development of PCMs has been carried out using both inorganic compounds such as hydrated salts and organic compounds such as paraffin, fatty acids, polyethylene glycol (PEG), and polymeric materials [315,316]. PCMs are classified according to their transformation properties, namely solid–liquid (melting), liquid–solid (freezing), liquid–gas (vaporization), solid–gas (sublimation), and gas–liquid (condensation) [317]. However, solid–liquid and solid–solid PCMs are commonly used in several applications. Besides these classifications, PCMs can be further classified into three categories based on temperature range, namely low-temperature PCMs (<15°C), intermediate temperature PCMs (15–90°C), and high-temperature PCMs (>90 °C) [318]. The intermediate temperature PCMs are most popular, used in solar and energy-saving application in concrete buildings [319,320]. The following three techniques for the use of PCM incorporation in concrete have been implemented: immersion (concrete immersion in melted PCMs) [321,322]; impregnation (impregnation of PCM in aggregates) [323,324]; and direct mixing (PCM directly mixed during the concrete casting) [325]. There are problems with the incorporation of PCMs (e.g., paraffin) directly mixed into concrete; these should be encapsulated within a shell [326], as the sustainability of paraffin is questionable in an alkaline medium due to its loss of thermal properties. In addition, it can interrupt the hydration reaction of cement, reducing its strength and increasing its porosity [327,328,329]. Hence, the stability of PCMs in the alkaline medium is increased by using microencapsulation methods, which reduce the direct contact between PCMs and concrete materials and increase thermal storage [314,330,331].

Marrani and Madhkha have studied the thermal properties of microencapsulated PCMs admixed in sandwich concrete panels [332]. They reported that with microencapsulated PCM admixed in the concrete interior walls, surface temperature decreased by up to 5.4 °C, which is an important feature in reducing the energy consumption for cooling on the inside of buildings. Hunger et al. prepared concrete with micro-encapsulated PCM and explored the properties of fresh and hardened concrete [327]. They reported that the microencapsulated PCMs enhanced the thermal performance of concrete; however, they also reported a significant reduction in compressive strength. Due to the mixing process, the microcapsule PCMs were damaged and released paraffin into the cement matrix. Cabeza et al., prepared a small house-sized concrete building with and without PCMs and examined the heat storage performance of PCM concrete compared to standard concrete. Based on the experimental results, they found the temperature with and without PCM concrete reached 36 °C and 39 °C, respectively [330]. Han et al. prepared concrete building models with and without PCMs, and examined the results in both laboratory and outdoor conditions [333]. They found a temperature difference of up to 6.8°C between the interior and outside surfaces of the building models made with concrete with and without PCMs. Rena et al. have reported that microencapsulated PCM admixed concrete improved thermal storage performance, and that its storage performance was directly proportional to the increasing addition of microencapsulated PCMs in concrete [334]. The surface temperature of PCM admixed concrete was reduced to 3.9 °C compared to the control mix. The microencapsulated PCMs can effectively absorb and release heat; thus, PCM concrete (self-temperature adjusting) has great potential application for reducing the consumption of energy in hot climates. However, limited studies are available in this area [310]; hence, continuous research and motivation are required to develop the use of PCMs in concrete structures without affecting the traditional properties of concrete. In addition, achieving passive cooling [335] and long-term stability with microencapsulated PCMs in concrete requires further investigation in order to realize its potential application in real concrete structures.

## 4. Conclusions

The following conclusions can be drawn from the present review:

- SCMs such as fly ash at levels of up to 25 to 30% can be safely used to replace Portland cement, resulting in better properties. A significant level of replacement of Portland cement by fly ash is not only beneficial to the concrete but also minimizes the production of cement, and thereby reduces the greenhouse gas effect as per the following equation:
(11) $${\mathrm{CaCO}}_{3}\;\to \mathrm{CaO}+{\mathrm{CO}}_{2}$$
- Mechanical grinding, thermal activation, and chemical activation accelerates the hydration reaction of fly ash with cement. Activation of fly ash is essential in order to obtain better performance in both mechanical and corrosion-resistant properties. Activation methodology increases the number of connecting points in fly ash and binds with cement within a short period. Activated fly ash-admixed cement yield designs showed compressive strength within 28 days on par with ordinary Portland cement.
- GGBS at levels up to 50 to 55% can be safely used to replace Portland cement, with better properties. GGBS has undergone in situ activation, with other alkalis present in it. The active ingredients in GGBS are highly reactive; hence, it undergoes a hydration reaction with cement which forms bulky calcium silicate hydrate, leads to a pore blocking effect.
- Bagasse ash at levels up to 10–15% can be safely used to replace Portland cement, with better properties. The presence of amorphous silica in SBA reacts with free Ca(OH)_2_ in the cement matrix, which produces secondary C-S-H gel and improves the compressive strength of concrete. SBA contains Al_2_O_3_, which reacts with Ca(OH)_2_, leading to the formation of C-A-H, which can reduce sulphate attack. Unreacted silica in SBA acts as a pore filler, which can reduce porosity and voids in concrete, which is helpful for increasing resistance to chloride penetration and reducing the corrosion rate of steel rebar.
- The reaction of silica fume in concrete involves both a physical contribution and chemical action. As a physical contribution, adding silica fume fills the spaces in the interfacial region of cement grains. As a chemical contribution, silica fume contains more than 90% very highly amorphous SiO_2_. Thus, it is a highly reactive pozzolanic material and readily reacts with calcium hydroxide to form C-S-H gel and provide hardened concrete.
- RHA is an effective SCM, and at an optimal replacement level up to 15–20% of Portland cement improves strength and reduces the sulphate reaction and chloride diffusion, as well as the corrosion rate of embedded steel rebar. The pozzolanic reaction is favorable in RHA when reactive silica is in its amorphous state, leading to the formation of additional C-S-H gel, which can improve the strength and durability of concrete.
- SCM-blended concrete resists both chloride and sulphate attack. Hence, concrete with SCMs is highly useful to construct civil infrastructure such as bridges, nuclear reactors, ports, seashore and marine environment areas, etc.
- With the rapid growth of the automobile industry in the 21^st^ century, tire-derived fuel ash may find applications in the construction industry. TDFA contains 25–30% SiO_2_ and 30–35% CaO, and thus is considered a suitable pozzolanic material. Furthermore, TDFA can be used as a partial replacement in cement and also used as a coarse aggregate in concrete.
- The purpose of corrosion-inhibiting admixtures is to protect rebar from corrosion. They are added to the concrete during the casting stage itself. Hence, in the construction of any new concrete structures the use of corrosion inhibitors is inevitable. For an existing concrete structure, a migrating or electro-injection methodology may be adopted to safeguard the embedded steel in concrete.
- Self-curing concrete is essential in arid and semi-arid regions where scarcity of water is a critical problem. Self-curing concrete minimizes cracking and shrinkage in concrete, and thereby improves its durability. The biggest advantage of self-curing concrete is the elimination of labour requirements, bypassing the laborious curing process of 28 days (for OPC) or 90 days (for PPC) post-construction.
- The self-healing process in concrete can be achieved by autonomous healing, autogenous healing, and microbial methods. In all three, the concrete is rendered capable of repairing cracks automatically without any external inspection or human involvement. However, this methodology is limited to smaller cracks only.
- Superhydrophobic chemical admixtures can be applied to concrete either through surface coatings or by admixture in concrete. In both methods, hydrophobic materials resist water and offer protection to both concrete and rebar.
- Electromagnetic shielding concrete has applications in both military buildings and civil applications. EMI shielding can be achieved by either absorption or reflection. Carbon materials, special types of conducting polymers, and metal fibers play important roles in making EMI-shielded concrete.
- Self-temperature adjusting concretes are very useful in tropical countries because indoor concrete buildings consume more energy for cooling during the summer seasons. This can be achieved by utilising phase changed materials (PCMs) in the concrete and thereby mitigating thermal effects.
- The integration of innovative technologies such as nano, geopolymer, 3D printing/digital production, bio, self-assembly, and organic–inorganic copolymerization may soon align with existing technologies to promote the growth of multipurpose structures, and provide a boon to the construction industry.

## Figures and Tables

**Table 1:** Chemical composition of fly ash and source data from [29].

Fly Ash	Chemical Compounds (wt%)								
	SiO_2_	Al_2_O_3_	Fe_2_O_3_	CaO	MgO	SO_3_	Na_2_O	K_2_O	LOI
Class C	20.7	9.01	32.0	27.1	2.05	1.61	1.00	2.51	2.97
Class F	55.23	25.95	10.17	1.32	0.31	0.18	1.59	1.59	5.25

**Table 2:** Chemical composition of GGBS.

Chemical Compounds (wt%)									Physical Properties	
SiO_2_	Al_2_O_3_	Fe_2_O_3_	CaO	MgO	SO_3_	Na_2_O	K_2_O	LOI	Specific Gravity	Blaine (cm^2^/g)
10.38–27.09	3.24–15.54	0.2–43.14	35.43–43.87	1.03–2.06	0.07–1.35	0.19–0.49	0.09–1.82	0.20–0.86	2.95	4603

**Table 3:** Chemical composition of silica fume.

References	Chemical Compounds (wt%)									Physical Properties	
	SiO_2_	Al_2_O_3_	Fe_2_O_3_	CaO	MgO	SO_3_	Na_2_O	K_2_O	LOI	Specific Gravity	Blaine (cm^2^/g)
[65]	95.7	0.2	0.1	0.4	0.5	-	0.2	0.7	1.99	2.22	19,000
[66]	95.0	1.7	2.0	-	0.9	-	0.2	1.02		2.21	14,000
[67]	91.9	1.05	1.11	1.35	0.61	-	0.6	1.73	1.27	2.30	-
[68]	93.6	0.8	0.5	1.8	1.10	-	0.1	0.1	1.0	-	-
[69]	96.0	1.1	1.45	1.2	0.18	0.23	0.45	1.2	-	2.15	17,800
[70]	93.0	0.58	2.79	0.60	1.00	0.5	1.0	0.1	0.5	2.20	2920

**Table 4:** Chemical composition of RHA.

References	Chemical Compounds (wt%)									Physical Properties	
	SiO_2_	Al_2_O_3_	Fe_2_O_3_	CaO	MgO	SO_3_	Na_2_O	K_2_O	LOI	Specific Gravity	Blaine (cm^2^/g)
[85]	92.95	0.31	0.26	0.53	0.55	-	0.08	2.06	1.97	-	-
[92]	93.2	0.4	0.1	1.1	0.1	0.9	0.1	1.3	3.7	2.23	11200
[94]	90.21	2.12	0.8	1.27	0.67	-	0.14	0.76	1.56	-	-
[100]	86	0.2	1.85	4.81	4.5	1.18	1.14	3.68	8.55	2.3	-
[101]	95.04	0.3	0.44	1.25	0.45	0.01	0.09	1.40	0.51	2.1	-
[102]	88.32	0.46	0.41	0.67	0.44	0.08	0.12	2.91	5.81	2.11	-

**Table 5:** Chemical composition of SBA.

References	Chemical Compounds (wt%)									Physical Properties	
	SiO_2_	Al_2_O_3_	Fe_2_O_3_	CaO	MgO	SO_3_	Na_2_O	K_2_O	LOI	Specific Gravity	Blaine (cm^2^/g)
[113]	63.1	7.56	4.59	8.28	4.54	1.92	1.24	5.43	4.10	3.78	4946
[115]	72.95	1.68	1.89	7.77	1.98	4.45	-	9.28	0.21	1.91	1450
[116]	63.0	31.5	1.79	0.48	0.39	-	-	-	0.714	2.2	25000
[117]	77.25	6.37	4.21	4.05	2.61	0.11	1.38	2.34	2.47	-	-
[118]	63.16	9.70	5.40	8.40	2.90	2.87	-	-	6.90	-	-
[119]	65.0	4.8	0.9	3.9	-	0.9	-	2.0	10.5	2.24	12500
[120]	84.16	1.68	4.40	0.36	0.15	1.93	0.18	0.57	6.04	-	-
[121]	80.8	5.1	1.6	3.1	0.3	1.5	0.8	6.3	0.4	-	-
[122]	88.2	2.3	5.1	0.6	0.4	0.1	0.1	1.3	1.75	-	-

**Table 6:** Chemical composition of TDFA.

References	Chemical Compounds (wt%)									Physical Properties	
	SiO_2_	Al_2_O_3_	Fe_2_O_3_	CaO	MgO	SO_3_	Na_2_O	K_2_O	C	Specific Gravity	Blaine (cm^2^/g)
[141]	25.4	4.03	5.59	36.4	-		0.57	0.76	3.21	-	-
[142]	27.5–31.1	6.49–14.5	-	24.6–35.9	-	5.14–10.6	-	-	-	-	5200

**Table 7:** Corrosion inhibiting admixtures in concrete.

Exposure Conditions	Type of Inhibitor	Name of Inhibitor	Dosage of Inhibitor	Aggressive Conditions/Dosage	Efficiency (%)	Reference
Ternary cement extract	Anodic/ Inorganic	Calcium nitrite	0%	1% of Cl^-^	-	[198]
				2% of Cl^-^	-	
				3% of Cl^-^	-	
			0.5%	1% of Cl^-^	91	
				2% of Cl^-^	89	
				3% of Cl^-^	81	
Portland pozzolona Cement extract		Sodium hydroxide	0 wt % of cement	30,000 ppm Cl^−^	-	[193]
			1 wt % of cement		7.47	
		Sodium hydroxide + sodium citrate	1 wt % of cement	30,000 ppm Cl^−^	12.52	
		Sodium hydroxide + sodium citrate + sodium stannate			33.43	
		Sodium hydroxide + sodium citrate + sodium stannate + CaO			45.15	
Sat. Ca(OH)_2_		Lithium nitrite	8.94(g/L)	0.99 NaCl(g/L)	85.75	[199]
			17.21(g/L)		83.26	
Concrete medium		NaNO_2_	0%	3% NaCl	-	[157]
			1%		87.9	
			2%		87.1	
			3%		85.83	
		ZnO	1%		93.75	
			2%		94.16	
			3%		91.66	
		NaNO_2_ + ZnO	1%		95.83	
			2%		94.58	
			3%		93.75	
SCPS		NaNO_2_	1500 ppm	3.5% NaCl	55%	[200]
		Trisodium Citrate	150 ppm		72.5	
		Zinc Acetate	50 ppm		55	
		Zinc Acetate + Trisodium citrate	50 ppm + 100 ppm		78	
Cement mortar	Anodic/ Inorganic	Sodium nitrate	0.4 mol per 1 kg cement	3.5% NaCl (360 days)	21.4	[201]
	Cathodic/ Inorganic	Sodium phosphate			7.5	
		Sodium phosphate	7% (by wt. of cement)	1% of Cl^-^ (by wt. of cement) (360 days)	97.7	[202]
Carbonated concrete solutions (0.0315 mol/L)		DiSodium Hydrogen Phosphate	20 mmol/L	0.1 mol/L NaCl	52	[203]
			60 mmol/L		93	
			100 mmol/L		99.95	
Chloride contaminated cement mortar(3% of NaCl (wt%))		Sodiumpyro phosphate	0%	-	-	[204]
			0.3%	-	78	
			0.6%	-	89	
			1.2%	-	58	
			2.4%	-	46	
Cement Concrete	Organic	Monoethanolamine	1%	exposed to 3% NaCl	49.58	[157]
			2%		50.8	
			3%		38.75	
		Diethonolamine	1%	exposed to 3% NaCl	35.42	
			2%		28.33	
			3%		39.16	
		Triethonalamine	1%	exposed to 3% NaCl	77.04	
			2%		62.5	
			3%		53.33	
Sat.Ca(OH)_2_	Organic	Deoxyribonucleic acid	0.0050 %	0.01 mol/L NaCl add every day (7 Days)	58.60	[205]
		Dicyclohexyl ammonium nitrite	Sat.	0.1 M NaCl	97	
		5-Hexyl-benzotriazole	0.005 M	0.1 M NaCl	67	
		Sodium β-glycerophosphate	0.05 M	0.1 M NaCl	92	
Cement Mortar	Organic	Dicyclohexyl ammonium nitrite	0.5(In/Cl^-^ ratio)	0.02 Cl^−^/cement wt. ratio	88	[205]
		Sodium β-glycerophosphate			90	
Carbonated alkali-activated fly ash mortar	Organic	Disodium β-glycerol phosphate Pentahydrate + sodium 3-aminobenzoate	0.05 M + 0.05 M	Immersed in 1% NaCl	63	[206]
		Disodium β-glycerol phosphate pentahydrate + sodium N-phenylanthranilate	0.05 M + saturated		81	

**Table 8:** Superhydrophobic additives admixed in concrete.

S.No	Method	Hydrophobic Material	Water Contact Angle (°)	Sliding Angle (°)	Reference
1	Admixed	Fluoroalkylsilane	158	6.1 ± 1.2	[253]
2	Admixed	1H,1H,2H,2H-Perfluorodecyltriethoxysilane	162	-	[255]
3	Admixed	Stearic acid with GGBS	155.7	-	[261]
4	Admixed	Stearic acid emulsion	130	-	[262]
5	Admixed	Polydimethoysilane	140	-	[263]
6	Admixed	Tire rubber	100–120	-	[264]
7	Concrete immersed	Steric acid + alcohol	167.2	4.2	[265]

**Table 9:** Electromagnetic shielding effectiveness of concrete with various chemical admixtures.

EMI Shielding Materials	Thickness of Specimen	Shielding Efficiency (SE)	Frequency Range	Reference
Coke (9.18%)	0.48 cm	49–51	1.0–1.5 GHz	[266]
Carbon black	1.0 cm	6–8 dB	2–8 GHz	[274]
Carbon Black	3.0 cm	20 dB and 10 dB	8.0–18.0 GHz and 18–26.5 GHz	[281]
Graphite	0.3 cm	10–40 dB	200–1600 MHz	[275]
Colloidal Graphite	0.44 cm	22.3 dB and 25.6 dB	1.0 and 1.5 GHz	[294]
Carbon Fiber (0.1µm diameter)/	0.41 cm	28.7–30.2 dB	1.0–2.0 GHz	[295]
Graphite and Carbon Fiber (0.1µm diameter)	-	19.8 dB	1.0 GHz	[296]
Graphite fine powder	2.0	2.4 dB	50–400 MHz	[297]
Carbon fiber (CF)	0.7 cm	12.5 dB to 4.9 dB	2.0–18.0 GHz	[298]
Graphene oxide (30%) with ferrofluid	10 cm & 30 cm	12 dB & 80 dB	2.6 GHz	[299]
CNT	5 cm	60–80 dB	1.7–2.6 GHz	[290]
MWCNT	3.0 cm	15 dB & 30 dB	2 GHz & 8 GHz	[300]
MWCNT	-	27 dB	8.2–12.4 GHz	[301]
Steel fiber	-	70 dB	1.5 GHz	[279]
Steel fiber/CF/PVA fiber	3.0 cm	20–40 dB	8–18 GHz	[302]
Mn-Zn ferrite	1.0 cm	15 dB	12 GHz	[283]
Natural Magnetite content	0.5 cm	10 dB & −28 dB	0.8 GHz & 3.7 GHz	[303]
Copper slag	-	7–8 dB	500–1.5 GHz	[304]
Fe_3_O_4_	0.7 cm	8.2–12.4 GHz	20–27 dB	[271]
TiO_2_	1.0 cm	8–18 GHz	−7.5 dB	[285]
Nickel fiber	0.6 cm	1–1500 MHz	19.85–24.48 dB	[305]

## Data Availability

The raw/processed data required to reproduce these findings cannot be shared at this time as the data also forms part of an ongoing study.
